# High resolution clustering of *Salmonella enterica *serovar Montevideo strains using a next-generation sequencing approach

**DOI:** 10.1186/1471-2164-13-32

**Published:** 2012-01-19

**Authors:** Marc W Allard, Yan Luo, Errol Strain, Cong Li, Christine E Keys, Insook Son, Robert Stones, Steven M Musser, Eric W Brown

**Affiliations:** 1 Office of Regulatory Science, Center for Food Safety & Applied Nutrition, U.S. Food & Drug Administration, 5100 Paint Branch Parkway, College Park, MD 20740, USA; 2 Office of Food Defense, Communications, and Emergency Response, Center for Food Safety & Applied Nutrition, U.S. Food & Drug Administration, 5100 Paint Branch Parkway, College Park, MD 20740, USA; 3 Food & Environment Research Agency, Sand Hutton, York, YO41 1LZ, UK

## Abstract

**Background:**

Next-Generation Sequencing (NGS) is increasingly being used as a molecular epidemiologic tool for discerning ancestry and traceback of the most complicated, difficult to resolve bacterial pathogens. Making a linkage between possible food sources and clinical isolates requires distinguishing the suspected pathogen from an environmental background and placing the variation observed into the wider context of variation occurring within a serovar and among other closely related foodborne pathogens. Equally important is the need to validate these high resolution molecular tools for use in molecular epidemiologic traceback. Such efforts include the examination of strain cluster stability as well as the cumulative genetic effects of sub-culturing on these clusters. Numerous isolates of *S*. Montevideo were shot-gun sequenced including diverse lineage representatives as well as numerous replicate clones to determine how much variability is due to bias, sequencing error, and or the culturing of isolates. All new draft genomes were compared to 34 *S*. Montevideo isolates previously published during an NGS-based molecular epidemiological case study.

**Results:**

Intraserovar lineages of *S*. Montevideo differ by thousands of SNPs, that are only slightly less than the number of SNPs observed between *S*. Montevideo and other distinct serovars. Much less variability was discovered within an individual *S*. Montevideo clade implicated in a recent foodborne outbreak as well as among individual NGS replicates. These findings were similar to previous reports documenting homopolymeric and deletion error rates with the Roche 454 GS Titanium technology. In no case, however, did variability associated with sequencing methods or sample preparations create inconsistencies with our current phylogenetic results or the subsequent molecular epidemiological evidence gleaned from these data.

**Conclusions:**

Implementation of a validated pipeline for NGS data acquisition and analysis provides highly reproducible results that are stable and predictable for molecular epidemiological applications. When draft genomes are collected at 15×-20× coverage and passed through a quality filter as part of a data analysis pipeline, including sub-passaged replicates defined by a few SNPs, they can be accurately placed in a phylogenetic context. This reproducibility applies to all levels within and between serovars of *Salmonella *suggesting that investigators using these methods can have confidence in their conclusions.

## Background

Foodborne pathogens cause an estimated 9.4 million human illnesses in the U.S. each year, resulting in nearly 60,000 hospitalizations and over 1,300 deaths [1-4]. *Salmonella enterica *remains one of the most devastating of these foodborne pathogens with 11% of all food related deaths being attributed from exposure to this bacterium [4]. The genus *Salmonella *comprises two species, *S. enterica *and *S. bongori*, both of which have been found in the food supply. Six subspecies of *S. enterica *have been described (I-IIIa, IIIb, IV, and VI) that can be found in a variety of mammalian and non-mammalian hosts including humans, cattle, birds, turtles, and snakes. Most non-typhoidal salmonellosis cases in mammals, including humans, come from over 1700 different *Salmonella *group (subspecies) I serovars. While several group I serovars such as *S*. Typhimurium and *S*. Enteritidis have been studied more widely, the genetic and phylogenetic diversity defining many of the important group I *Salmonella*e remains poorly understood.

One of these serovars, *Salmonella enterica *subsp. *enterica *serovar Montevideo (*i.e., S*. Montevideo) is one of the top ten most common serovars associated with contaminated foods. This serovar was recently associated with a Pistachio recall in 2008, and more recently, with contamination of certain pet treats http://www.fda.gov/Safety/Recalls/ucm218039.htm. Moreover, this serovar has been implicated in contamination events involving numerous meat and cheese products http://www.outbreakdatabase.com/site/search/?tag=s.+montevideo. More recently, a strain of *S*. Montevideo was linked to more than 240 illnesses in 38 states after being found in red and black pepper used in the production of contaminated Italian-style spiced meats [[5], http://www.cdc.gov/Salmonella/montevideo/montevideo_timeline2.pdf]. It is important to note that many of these highly clonal strains of *S*. Montevideo often confound epidemiological investigations because pulsed-field gel electrophoresis (PFGE) is unable to always distinguish outbreak-related strains from other genetically similar strains unassociated with the same outbreak. Strains of this nature often retain common PFGE patterns despite their sporadic or more historic origins.

The accurate subtyping and subsequent clustering of isolates of a bacterium associated with a foodborne outbreak event is essential for successful investigation and eventual traceback to a specific food or environmental source [6-12]. In this regard, PFGE continues to deliver useful genetic typing information by facilitating public health investigations for nearly two decades. In certain cases, however, highly clonal strains, common among some group I *Salmonella*e, confound epidemiological investigations because PFGE provides limited genetic differentiation of these strains. That is this approach often lacks the resolution for differentiating highly clonal bacterial isolates. In response to such events, federal public health and food safety laboratories are exploring next-generation sequencing (NGS) to define complex outbreak scenarios. NGS refers to highly parallel robotic genomic sequencers, like Roche 454 GS Titanium technology, that are being used to accomplish the whole genome sequencing (WGS) of a bacterial pathogen.

NGS is contributing long anticipated solutions to what were once viewed as insurmountable challenges, in the genetic analysis of bacterial pathogens [13-16]. Complete genome sequences from multiple bacterial strains can now be collected and analyzed in just a few days [17], underscoring the future potential of this technology as a molecular epidemiological tool to assist in foodborne outbreak investigations. Recent examples in the literature illustrate the ability of NGS to discern the high-resolution genetic relatedness and unrelatedness of otherwise indistinguishable isolates based on the microevolutionary genetic change that define clinical isolates, outbreak isolates found in foods, and their environmental counterparts [18-20].

These novel applications of NGS are buttressed by a massive influx of new genomic data, producing new discoveries about the critical genes that define particular pathogens, and important genomic changes associated with pathogenicity, antibiotic resistance, and unique carbon source usage [19,20]. However, the race to sequence more bacterial pathogen genomes must be tempered by the realities and rigor of formal methods validation processes for tools deployed in epidemiological investigation http://www.fda.gov/MedicalDevices/DeviceRegulationandGuidance/GuidanceDocuments/ucm077862.htm. This validation process is not only required for regulatory action by federal and state laboratories whose duty it is to conduct these tests, but these general procedures must be applied if the technology is to meet scientific admissibility requirements in a legal setting. Although still being developed, historical paradigms exist for the validation of sequence data. Capillary electrophoresis (CE) sequencing, for example, has been a standard technology since the early 1990s, and its accuracy has proven to be sufficient so that CE is now widely applied by a variety of federal agencies engaged in activities spanning forensic and molecular epidemiologic analyses [21,22].

Herein, we demonstrate the value of NGS in defining the diversity of *Salmonella *Montevideo using a representative set of environmental, laboratory, food, and clinical strains, some of which have been associated repeatedly with contamination events in several food sources [5]. Here, our analyses using NGS data provided far greater resolving power than previously available from other techniques. This information was essential to reconstructing both the deep phyletic relationships of this serovar and terminal relationships among highly clonal *S. Montevideo *isolates. Moreover, the clonally derived outbreak cluster of *S*. Montevideo were defined by a few single nucleotide polymorphisms (SNPs) while more geographically or temporally removed isolates showed tens to thousands of SNP differences. Additionally, NGS technology revealed considerable genomic stability and high reproducibility for SNP targets used in the clustering of closely related isolates within an important and emerging serovar of *Salmonella enterica*.

## Results

### NGS reveals substantial intra-serovar diversity within *Salmonella *Montevideo

**Figure 1:**
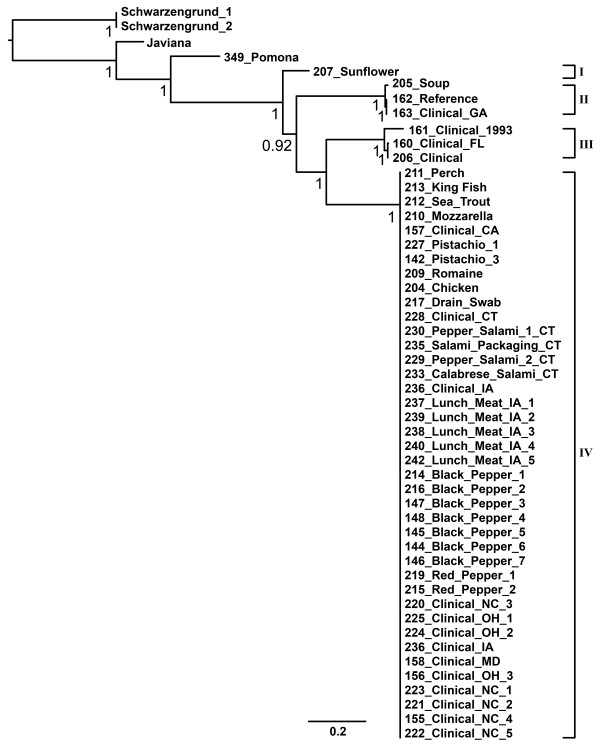
**Phylogenetic diversity of *Salmonella *Montevideo based on a GARLI analysis of 72,063 variable SNP sites of which 63,987 were identified as parsimony informative**. The tree was rooted with four outgroups including *S*. Schwarzengrund, *S*. Pomona, and *S*. Javiana. Terminal names correspond to samples in Table 1. The numbers at the base of each node are bootstrap scores with most of the deepest nodes supported at 100%. The scale bar units are nucleotide substitutions per site and these are proportional across the branch lengths with longer branches having greater substitutions. *S*. Montevideo strains partitioned into four clades designated I-IV.

In order to explore the evolutionary genetic diversity of *Salmonella *Montevideo, NGS analysis was performed on 47 strains of this serovar (Table 1). This included assembling the raw reads to form contigs of overlapping sequence, annotating those contigs to determine which genes were present, and then determining homology among genes and aligning and concatenating those genetic elements for population and phylogenetic analyses. Roche-Titanium whole-genome shotgun sequencing technology [23,24] provided 15-20× coverage for each genome reported, and downstream contig assembly and sequence alignment provided over 4.5-5 mbp of assembled contigs for each isolate. Additional data filtering yielded 72,063 variable SNP sites of which 63,987 were identified as parsimony informative (*i.e.*, SNPs shared by two or more strains in the alignment) and subjected to phylogenetic analysis on the FDA bioinformatics, Linux based computer cluster using likelihood and parsimony methods. The resultant evolutionary tree derived from the informative SNP data yielded two important observations (Figure 1). First, *S*. Montevideo formed a monophyletic group of strains phylogenetically distinct from other neighboring serovars including *S*. Schwarzengrund, *S*. Pomona, and *S*. Javiana. Second, *S*. Montevideo strains partitioned into four disparate clades (designated I-IV), several of which were defined by a mixture of both natural and laboratory isolates. Clade III, for example, comprised a clinical isolate associated with tomato (206_Clinical) as well as a single strain (160_Clinical_FL) from the widely characterized subspecies I *Salmonella *Reference collection, SARB [25].

**Table 1:** List of isolates sequenced for comparison.

FDA Name	Tree Label	Locus Tag	GenBank	SRA	NCBI BioProject	Biosample	Full Name
142	142_Pistachio_3	SEEM315	AESH00000000	SRX101634, SRX118696, SRX119982	46535	710595	Salmonella enterica subsp. enterica serovar Montevideo str. 315996572
144	144_Black_Pepper_6	SEEM971	AESI00000000	SRX101636, SRX119983, SRX118697	46539	710606	Salmonella enterica subsp. enterica serovar Montevideo str. 495297-1
145	145_Black_Pepper_5	SEEM973	AESJ00000000	SRX101642	46541	710617	Salmonella enterica subsp. enterica serovar Montevideo str. 495297-3
146	146_Black_Pepper_7	SEEM974	AESK00000000	SRX101643	46543	710624	Salmonella enterica subsp. enterica serovar Montevideo str. 495297-4
147	147_Black_Pepper_3	SEEM201	AESL00000000	SRX101644	46545	710625	Salmonella enterica subsp. enterica serovar Montevideo str. 515920-1
148	148_Black_Pepper_4	SEEM202	AESM00000000	SRX101645, SRX118768	46547	710626	Salmonella enterica subsp. enterica serovar Montevideo str. 515920-2
155	155_ Clinical_NC_4	SEEM054	AESO00000000	SRX101647, SRX118769	46903	710628	Salmonella enterica subsp. enterica serovar Montevideo str. NC_MB110209-0054
156	156_Clinical_OH_3	SEEM675	AESP00000000	SRX101648, SRX119984, SRX118770	46905	710629	Salmonella enterica subsp. enterica serovar Montevideo str. OH_2009072675
157	157_Clinical_CA	SEEM965	AESQ00000000	SRX101649, SRX119443, SRX118771	46907	710596	Salmonella enterica subsp. enterica serovar Montevideo str. CASC_09SCPH15965
158	158_Clinical_MD	SEEM507	AETA00000000	SRX101650	49405	710597	Salmonella enterica subsp. enterica serovar Montevideo str. MD_MDA09249507
160	160_Clinical_FL*	SEEM031	AESR00000000	SRX105725	46911	754243	Salmonella enterica subsp. enterica serovar Montevideo str. SARB31
161	161_Clinical_1993*	SEEM710	AESS00000000	SRX105759	46913	754295	Salmonella enterica subsp. enterica serovar Montevideo str. ATCC BAA710
162	162_Reference*	SEEM010	AEST00000000	SRX105760	46915	754296	Salmonella enterica subsp. enterica serovar Montevideo str. LQC 10
163	163_Clinical_GA*	SEEM030	AESU00000000	SRX105761	46917	754297	Salmonella enterica subsp. enterica serovar Montevideo str. SARB30
204	204_Chicken	SEEM19N	AESV00000000	SRX101465, SRX118774	48457	710598	Salmonella enterica subsp. enterica serovar Montevideo str. 19N
205	205_Soup*	SEEM29N	AESW00000000	SRX105762	48459	754298	Salmonella enterica subsp. enterica serovar Montevideo str. 29N
206	206_Clinical*	SEEM42N	AESX00000000	SRX105763	48461	754299	Salmonella enterica subsp. enterica serovar Montevideo str. 42N
207	207_Sunflower*	SEEM41H	AESY00000000	SRX105764, SRX105765	49127	754300	Salmonella enterica subsp. enterica serovar Montevideo str. 4441 H
209	209_Romaine	SEEM801	AESZ00000000	SRX101467	49129	710599	Salmonella enterica subsp. enterica serovar Montevideo str. 81038-01
210	210_Mozzarella	SEEM877	AETB00000000	SRX101651	49987	710600	Salmonella enterica subsp. enterica serovar Montevideo str. 414877
211	211_ Perch	SEEM867	AETC00000000	SRX101652, SRX118775	49989	710601	Salmonella enterica subsp. enterica serovar Montevideo str. 366867
212	212_Sea_Trout	SEEM180	AETD00000000	SRX101653, SRX119985, SRX118776	49991	710602	Salmonella enterica subsp. enterica serovar Montevideo str. 413180
213	213_King Fish	SEEM600	AETE00000000	SRX101659	49993	710603	Salmonella enterica subsp. enterica serovar Montevideo str. 446600
214	214_Black_Pepper_1	SEEM581	AETF00000000	SRX101660	49995	710604	Salmonella enterica subsp. enterica serovar Montevideo str. 609458-1
215	215_Red_Pepper_2	SEEM501	AETG00000000	SRX101661, SRX119986, SRX118783	49997	710605	Salmonella enterica subsp. enterica serovar Montevideo str. 556150-1
216	216_Black_Pepper_2	SEEM460	AETH00000000	SRX101666	50021	710607	Salmonella enterica subsp. enterica serovar Montevideo str. 609460
217	217_Drain_Swab	SEEM020	AETI00000000	SRX103943, SRX103942, SRX118784, SRX119444	50023	710608	Salmonella enterica subsp. enterica serovar Montevideo str. 507440-20
219	219_Red_Pepper_1	SEEM6152	AETJ00000000	SRX103944	51379	710609	Salmonella enterica subsp. enterica serovar Montevideo str. 556152
220	220_Clinical_NC_3	SEEM0077	AETK00000000	SRX103945	51381	710610	Salmonella enterica subsp. enterica serovar Montevideo str. MB101509-0077
221	221_Clinical_NC_2	SEEM0047	AETL00000000	SRX103946, SRX118785, SRX119987	51383	710611	Salmonella enterica subsp. enterica serovar Montevideo str. MB102109-0047
222	222_ Clinical_NC_5	SEEM0055	AETM00000000	SRX103951, SRX119988, SRX118786	51385	710612	Salmonella enterica subsp. enterica serovar Montevideo str. MB110209-0055
223	223_Clinical_NC_1	SEEM0052	AETN00000000	SRX103952, SRX119989, SRX118787	51387	710613	Salmonella enterica subsp. enterica serovar Montevideo str. MB111609-0052
224	224_Clinical_OH_2	SEEM3312	AETO00000000	SRX103953	51389	710614	Salmonella enterica subsp. enterica serovar Montevideo str. 2009083312
225	225_Clinical_OH_1	SEEM5258	AETP00000000	SRX103954	51391	710615	Salmonella enterica subsp. enterica serovar Montevideo str. 2009085258
227	227_Pistachio_1	SEEM1156	AETQ00000000	SRX103955, SRX118788	51393	710616	Salmonella enterica subsp. enterica serovar Montevideo str. 315731156
228	228_Clinical_CT*	SEEM5278	AHHS00000000	SRX105767	62845	754302	Salmonella enterica subsp. enterica serovar Montevideo str. CT_02035278
229	229_Pepper_Salami_2_CT*	SEEM5318	AHHT00000000	SRX105768, SRX118789	62847	754303	Salmonella enterica subsp. enterica serovar Montevideo str. CT_02035318
230	230_Pepper_Salami_1_CT*	SEEM5320	AHHU00000000	SRX105769, SRX119990, SRX118790	62849	754304	Salmonella enterica subsp. enterica serovar Montevideo str. CT_02035320
233	233_Calabrese_Salami_CT*	SEEM5321	AHHV00000000	SRX105770, SRX118791	51967	754305	Salmonella enterica subsp. enterica serovar Montevideo str. CT_02035321
235	235_ Salami_Packaging_CT*	SEEM5327	AHHW00000000	SRX105771	51973		Salmonella enterica subsp. enterica serovar Montevideo str. CT_02035327
236	236_Clinical_IA	SEEM9199	AETR00000000	SRX105772	51975	710618	Salmonella enterica subsp. enterica serovar Montevideo str. IA_2009159199
237	237_Lunch_Meat_IA_1	SEEM8282	AETS00000000	SRX103956, SRX118793, SRX119445	51979	710619	Salmonella enterica subsp. enterica serovar Montevideo str. IA_2010008282
238	238_Lunch_Meat_IA_3	SEEM8283	AETT00000000	SRX103957, SRX118793,	51981	710620	Salmonella enterica subsp. enterica serovar Montevideo str. IA_2010008283
239	239_Lunch_Meat_IA_2	SEEM8284	AETU00000000	SRX103958	51983	710621	Salmonella enterica subsp. enterica serovar Montevideo str. IA_2010008284
240	240_Lunch_Meat_IA_4	SEEM8285	AETV00000000	SRX103959	51985	710622	Salmonella enterica subsp. enterica serovar Montevideo str. IA_2010008285
241	241_Lunch_Meat_IA_6*	SEEM8286	NA	SRX105773, SRX105774	51987	754308	Salmonella enterica subsp. enterica serovar Montevideo str. IA_2010008286
242	242_Lunch_Meat_IA_5	SEEM8287	AETW00000000	SRX103960	51989	710623	Salmonella enterica subsp. enterica serovar Montevideo str. IA_2010008287
349	349_Pomona*	SEEPO729	AHIA00000000	SRX105896	61431	754430	Salmonella enterica subsp. enterica serovar Pomona str. ATCC 10729
397	237_Colony_1*	resequence of FDA237 colony 1	NA	SRX105897	51979	710619	Salmonella enterica subsp. enterica serovar Montevideo str. IA_2010008282
398	237_Colony_2*	resequence of FDA237 colony 2	NA	SRX105898	51979	710619	Salmonella enterica subsp. enterica serovar Montevideo str. IA_2010008282
399	237_Colony_3*	resequence of FDA237 colony 3	NA	SRX105899	51979	710619	Salmonella enterica subsp. enterica serovar Montevideo str. IA_2010008282
400	237_Colony_4_Rep_1*	resequence of FDA237 colony 4	NA	SRX105900	51979	710619	Salmonella enterica subsp. enterica serovar Montevideo str. IA_2010008282
401	400_Colony_4_Rep_2*	resequence 1 of FDA237/FDA400 colony 4	NA	SRX105901	51979	710619	Salmonella enterica subsp. enterica serovar Montevideo str. IA_2010008282
402	400_Colony_4_Rep_3*	resequence 2 of FDA237/FDA400 colony 4	NA	SRX105902	51979	710619	Salmonella enterica subsp. enterica serovar Montevideo str. IA_2010008282
403	400_Colony_4_Rep_4*	resequence 3 of FDA237/FDA400 colony 4	NA	SRX105903	51979	710619	Salmonella enterica subsp. enterica serovar Montevideo str. IA_2010008282
515	237_1st_Round_Passage*	serial resequencing FDA237 plate 1st round	NA	SRX105904	51979	710619	Salmonella enterica subsp. enterica serovar Montevideo str. IA_2010008282
516	237_2nd_Round_Passage*	serial resequencing FDA237 plate 2nd round	NA	SRX105905	51979	710619	Salmonella enterica subsp. enterica serovar Montevideo str. IA_2010008282
517	237_3rd_Round_Passage*	serial resequencing FDA237 plate 3rd round	NA	SRX105906	51979	710619	Salmonella enterica subsp. enterica serovar Montevideo str. IA_2010008282
518	237_4th_Round_Passage*	serial resequencing FDA237 plate 4th round	NA	SRX105907	51979	710619	Salmonella enterica subsp. enterica serovar Montevideo str. IA_2010008282
NA	Schwarzengrund_1	SASA	CP001127				Salmonella enterica subsp. enterica serovar Schwarzengrund str. CVM19633
NA	Schwarzengrund_2	SASB	ABEJ01000001				Salmonella enterica subsp. enterica serovar Schwarzengrund str. SL480
NA	Javiana	SEJ	ABEH00000000				Salmonella enterica subsp. enterica serovar Javiana str. GA_MM04042433

Entries include the FDA sample number, a simplified tree label, locus tags as well as various identifiers from the Genome BioProjects of these draft genomes. New accession and short read archive numbers are noted by an asterisk in the column entitled "Tree Label". All other accession numbers were published previously [5].

**Figure 2:**
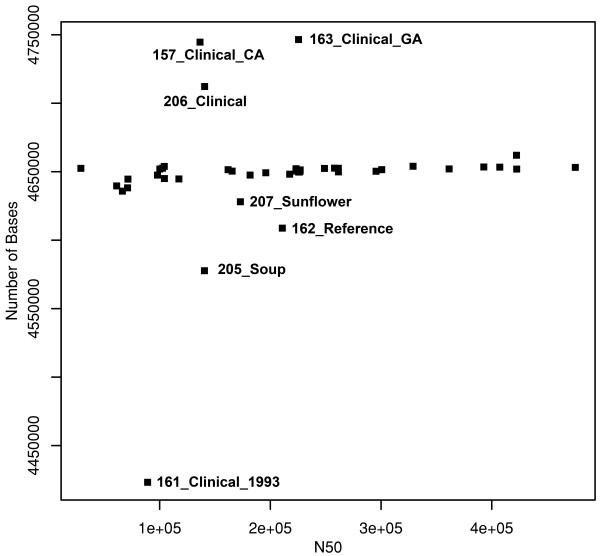
**Genome size variation and estimated N50 sizes within *Salmonella *Montevideo draft genome sequences**. The estimated N50 value is a rough estimate of the quality and coverage of the draft genomes which was sequenced to approximately 15-20× coverage. The N50 value represents the average contig size after assembly with the Newbler software. Isolate names correspond to samples in Table 1. Genome length ranged from less than 4.45 mbp to about 4.75 mbp, with most isolates approximately 4.65 mbp in size (unlabeled boxes). Only the larger or smaller genomes are listed.

Pairwise SNP variation between these four *S*. Montevideo lineages is listed in Table 2. Intra-serovar SNP diversity was remarkable among the four diverged *S*. Montevideo genome lineages ranging from 17,600 SNPs (clade I/clade II) to 23,800 SNPs (clade II/clade IV). This latter distance was astonishing given that SNP divergence between *S*. Montevideo lineage I and *S*. Pomona, a different group I serovar, falls well within this range (*i.e.*, 22,700 SNPs). In addition to the substantial SNP-based diversity noted among *S*. Montevideo lineages, genome size also fluctuated widely within this serovar (Figure 2). That is, genome length ranged from less than 4.45 million bp to about 4.75 million bp, sorting largely along intra-serovar clade divisions revealed in the phylogenetic tree (Figure 1). Most of the observed genome size differences appear to be due to the presence or absence of phage elements. The CA clinical isolate 157, for example, is bigger than the outbreak cluster in general due to phage D6. In addition, *S*. Montevideo strain 163 appears to be enlarged due to insertion of a plasmid pRA1, while strain 206 retains an uncharacterized phage-like sequence and elements of the SPI-7 pathogenicity island. Conversely, two smaller *S*. Montevideo genomes, 162 and 205, appear to be missing the putative *Salmonella *phage sequence relative to the outbreak cluster (i.e., clade IV, Figure 1). Akin to findings reported previously on the stress-induced acquisition and loss of phage elements in the *Salmonella *genome [26], these data signal an important role for insertions and deletions in the diversification of specific clones of *S*. Montevideo, and, taken together with the above SNP findings, point to a serovar of non-typhoidal *Salmonella *comprised of several genomically diverged and phylogenetically distinct clones [27-29].

**Table 2:** Pairwise distances (no. of nucleotide differences) and Standard Errors (SE) for the major groups shown in Figure 1.

	Schwarzengrund	Javiana	Pomona	Clade I	Clade II	Clade III	Clade IV
**Schwarzengrund**	23 (2.9)						
**Javiana**	25700 ( 70)	NA					
**Pomona**	27600 (130)	17800 ( 73)	NA				
**Clade I**	32500 ( 85)	23700 ( 92)	22700 (120)	NA			
**Clade II**	33000 ( 98)	27100 (110)	25900 ( 73)	17600 (120)	499 (8.5)		
**Clade III**	32800 (130)	26100 ( 59)	27700 (110)	18400 ( 78)	22200 ( 44)	2718 ( 43)	
**Clade IV**	34300 (150)	26500 (110)	24600 (150)	19300 (170)	23800 (130)	19300 (130)	13.5 (2.0)

Distances were calculated using the concatenated alignment of 63,987 informative SNPs that estimates the diversity between *S*. Schwarzendgrund, *S*. Javiana, *S*. Pomona and the major clades of *S*. Montevideo observed.

### NGS phylogenetically differentiates a clonal lineage of *Salmonella *Montevideo

**Figure 3:**
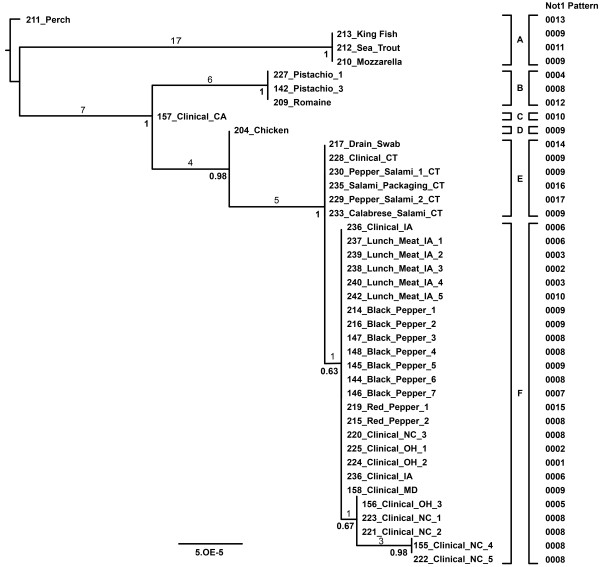
**Phylogenetic diversity and relationships among a single *S*. Montevideo clone**. GARLI phylogenetic analysis of the outbreak isolates was performed on a set of 43 concatenated ORFs containing informative SNPs (Table 3). Terminal names, scale bar, branch lengths and bootstrap scores are as in Figure 1. Numbers above the branches represent unique SNPs that define these internal branches. The phylogenetic analysis reported here partitions the *S*. Montevideo clone into 6 lineages (A-F) and expands upon a previous tree [5] with the inclusion of 5 more strains and the noted expansion of outbreak strains into clade E. To the right of the tree, each isolate is labeled with the *Not*1 pattern that was determined using PFGE with each unique number identifying a new *Not*1 pattern.

The importance of NGS in ascertaining high-resolution phylogenetic and molecular epidemiological histories of infectious outbreak clones of bacterial pathogens has recently been noted [18,20]. In the current study, NGS was applied for reconstructing the detailed evolutionary genetic structure of an individual clone of *S*. Montevideo that is largely indistinguishable using PFGE. Specifically, NGS analysis was applied to a set of *S*. Montevideo isolates either associated with or genetically homologous to a food contamination event of spiced Italian-style meats in the U.S. in 2009 and 2010 http://www.cdc.gov/Salmonella/montevideo/index.html. We reported previously on the success of NGS for distinguishing some of these isolates from other clonally related isolates unassociated with this spiced-meat *S*. Montevideo outbreak [5]. Herein, we combined the genomes of 34 highly homogeneous *S*. Montevideos from food, environmental, and clinical sources from this spiced-meat outbreak with 24 newly sequenced (~15X) *S*. Montevideo genomes derived from clinical-food matches associated with the same spiced-meat contamination event. As an important control, historical *S*. Montevideos from within this clone were included that retained multiple identical PFGE patterns to the spiced-meat outbreak strains and were isolated from a variety of foods unassociated with this outbreak such as pistachios, chicken, Italian cheese, and several fishes from Indo-China. It is important to note that all of the clinical isolates included here (Figure 3) were collected in association with the spiced-meat outbreak event.

Results from the phylogenetic analysis provided several important findings relevant to the phylogenetic differentiation of clonal *S*. Montevideo strains (Figure 3). First, in contrast to the serovar tree presented in Figure 1, SNP diversity within this highly clonal sub-lineage of *S*. Montevideo was markedly lower as expected, less than 500 informative SNPs defined the entire tree. However, the resultant likelihood tree partitioned this clone into six distinct groups of isolates that were separated from neighboring groups by less than 100 parsimony informative SNPs each. Additionally, isolates associated previously with the spiced-meat outbreak clustered together in a group separate and distinct from groups of closely related *S*. Montevideos unassociated with this contamination event (*e.g.*, pistachios/B, chicken/D, and fish/A). From a phylogenetic perspective, clades E and F appear to capture the scope of the outbreak. There are several reasons that support this partition. Clinical isolates (*i.e.*, CT clinical isolates) associate closely with a drain strain from the facility forming clade E (Figure 3) and from contaminated spices collected at the facility along with a host of clinicals from several states (*i.e.*, IA, MD, NC, and OH) nearly all of which were indistinguishable from the food isolates (clade F, Figure 3). It is also noteworthy that clade F retained a subgroup of NC isolates that were separated from the other food and clinical spiced-meat strains by just a few SNPs. However, these isolates are clear monophyletic members of clade F, one of the two outbreak clades, and may have emerged from the base of this clade through microevolutionary change. Whatever the final explanation, NGS analysis coupled with a comparative phylogenetic approach not only fully differentiated this clone of *S*. Montevideo, but also provided high resolution genetic information that effectively delimited the scope of the outbreak event, affirming its potential as a powerful tool for supporting molecular epidemiologic investigation of clonal outbreaks of non-typhoidal *Salmonella *[5].

SNP variation within the *S*. Montevideo spiced-meat clone was nearly two logs lower than what was noted for total intra-serovar diversity. Nevertheless, the signature SNPs that delineated these six subgroups (A-F) originated from various regions around the *S*. Montevideo genome and included a variety of genes assigned to diverse cellular functions including metabolism, DNA synthesis and repair, transport and uptake, virulence, and stress response. A list of 43 genes from which the SNPS that characterize *S*. Montevideo clade IV were derived is provided in Table 3. A representative SNP from each of these genes is also provided in the table along with the subgroup that it defines and its bp coordinates. Thirty of these genes were annotated previously with assigned names and functions; however, 13 additional regions that provided signature SNPs are hypothetical and, as such, are cross-referenced by locus tags only. It is notable that a partial and select set of SNPS from 25 of these 43 genes are non-synonymous, and of the 14 SNPs in Table 3 that cluster together two or more *S*. Montevideo subgroups in Figure 3, all but three are protein- altering in nature. These data are intriguing given an NGS report documenting positive selection among a significant subset of core genes in adapted *Salmonella enterica *serovars [30].

**Table 3:** 43 Variable genes found within a clonal lineage of *Salmonella *Montevideo.

Gene	LT Locus	Drain Locus	Nuc	AA	Position	Clade	Feature
lig	STM2427	SEEM020_00085	C/T	T	1974	b	DNA ligase, NAD-dependent
perM	STM2493	SEEM020_00410	C/T	A	1034	d,e,f	permease PerM
aroB	STM3486	SEEM020_01090	C/T	V/A	488	b,c,d,e,f	shikimate kinase I
yrfI	STM3498	SEEM020_01145	C/T	T/I	353	b,c,d,e,f	heat shock protein
gntK	STM3542	SEEM020_01330	G/A	H/Q	93	a1	1) gluconate transporter GntU, 2) shikimate kinase
dppA	STM3630	SEEM020_01925	C/T	H	282	a1	dipeptide transport protein
tolB	STM0748	SEEM020_01960	G/T	A/S	64	a1	tolB protein precursor
citG	STM0619	SEEM020_04239	C/T	R/C	181	b,c,d,e,f	triphosphoribosyl-dephospho-CoA synthase
citF	STM0621	SEEM020_04249	C/T	V/A	602	b,c,d,e,f	citrate lyase alpha chain
ydfZ	STM1509	SEEM020_04749	G/A	P	174	b	putative selenium-binding protein YdfZ
	STM1546	SEEM020_04939	C/T	L	1473	d,e,f	1) putative multidrug efflux protein, 2) hypothetical protein
	SeSA_A1664	SEEM020_05139	C/T	L	667	a1	LysR substrate binding domain protein
	STM1627	SEEM020_05529	C/T	T	543	a1	alcohol dehydrogenase class III
	STM1628	SEEM020_05534	T/G	L/R	155	a1	putative cytoplasmic protein
	STM1671	SEEM020_05759	A/C	V	122	a1	putative regulatory protein
	STM1856	SEEM020_06993	G/T	E/Stop	316		putative cytoplasmic protein
fliC	STM1959	SEEM020_07518	T/A	N/K	723	a1	phase 1 flagellin
uhpA	STM3790	SEEM020_08264	A/G	l	60	a1	1) sensor histidine kinase UhpB, 2)transcriptional regulatory protein UhpA
nuoL	STM2318	SEEM020_09061	C/T	F	291	f2	NADH dehydrogenase I
	STM4534	SEEM020_10120	C/T	A/V	14	b	putative transcriptional regulator
ytfG	STM4401	SEEM020_10825	C/T	S/F	503	f3	conserved hypothetical protein
yjeM	STM4345	SEEM020_11085	C/T	L	1179	a1	putative APC family amino-acid transport protein
	STM4261	SEEM020_11575	G/A	V/I	4684		putative inner membrane protein
araD	STM0101	SEEM020_12590	C/A	Q/K	466	b	L-ribulose-5-phosphate 4-epimerase
araB	STM0103	SEEM020_12600	G/A	L	702	e,f	L-ribulokinase
	STM3260	SEEM020_13557	G/A	V/M	70	b	PTS family galactitol-specific enzyme IIC
	SeD_A3648	SEEM020_13697	A/G	D/G	263	d,e,f	hypothetical protein
pduV	STM2056	SEEM020_14356	G/A	D/G	353	b,c,d,e,f	propanediol utilization protein
orf408	STM1382	SEEM020_15066	G/A	T/A	1096	a1	putative regulatory protein, deoR family
ydiA	STM1348	SEEM020_15231	C/T	F	126	b,c,d,e,f	putative inner membrane protein
envE	STM1242	SEEM020_15746	T/C	I/T	446	a1	EnvE
ycfX	STM1220	SEEM020_15941	C/T	G	24	d,e,f	N-acetylglucosamine kinase
	STM4097	SEEM020_16375	G/A	S/N	119	a1	putative outer membrane lipoprotein
uvrD	STM3951	SEEM020_17067	G/A	G/E	2111	f2	DNA helicase II
	STM2404	SEEM020_17529	G/T	A/S	394	b	putative chloride channel permease
recB	STM2994	SEEM020_17950	G/A	S/G	901	d,e,f	exodeoxyribonuclease V
stdB	STM3028	SEEM020_18130	C/T	L	2433	f2	putative outer membrane usher protein
yqjI	STM3215	SEEM020_19095	C/A	H/N	562	e,f	1) transcriptional regulator (PadR), 2) family methyl-accepting chemotaxis protein II
ybhK	STM0801	SEEM020_19330	G/A	L	618	a1	conserved hypothetical protein
	STM0818	SEEM020_19410	G/T	A/S	277	e,f	1) putative ABC-type multidrug transport system, 2) membrane permease predicted cation efflux pump
yliD	STM0851	SEEM020_19565	T/C	W/R	760		putative ABC transporter inner membrane component
invE	STM2897	SEEM020_21151	C/G	L/V	757	f	invasion protein
yejM	STM2228	SEEM020_21392	G/A	A/E	395	a1	putative hydrolase of alkaline phosphatase superfamily

Variable genes are listed by their Genbank abbreviated and full name (feature) and by the blast locus hit to either a reference isolate LT2 or the Drain swab isolate. A representative nucleotide change observed within each gene is listed as well as whether this caused an AA change and to which phylogenetic group it was associated with from Figure 3 (A-F). These SNPs were the most useful for the spiced meat outbreak investigation and will be useful for both targeted resequencing efforts and for rapid subtyping methods for traceback of future *S*. Montevideo investigation and diagnosis.

**Figure 4:**
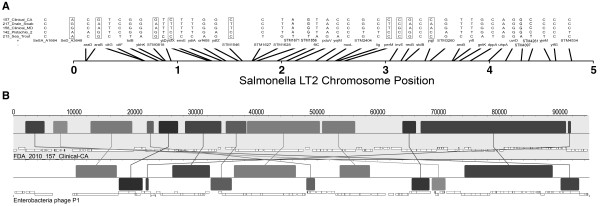
**NGS discovery of unique SNPs and insertional genetic attributes found in a highly homogeneous strain of S. Montevideo from California (157_Clinical_CA)**. (A) Isolate names correspond to samples in Table 1, and gene names correspond to the ORFs containing informative SNPs among a single *S*. Montevideo outbreak clone in Table 3. A representative nucleotide site observed across 5 isolates is listed for each ORF. ORFs are mapped against a reference of *S*. Typhimurium strain LT2 with lines going to approximate chromosomal positions relative to the reference (numbers in mbp). (B) A comparative MAUVE analysis of isolate 157_Clinical_CA revealed the presence of a 100 kb insertion with homology to Enterobacterial phage D6. Here we compared the isolate to another more complete homologous relative, phage P1 to document the insertion site. Graphic is standard MAUVE format showing putative genes as boxes with arrows documenting insertions and rearrangements. Forward and reverse strands are on opposite sides of the mid-line.

Although the majority of isolates composing the spiced-meat *S*. Montevideo clone generally exhibited a common genome length, one isolate from California (*S*. Montevideo 157_Clinical_CA) retained a noticeably larger genome than other members of this lineage (Figure 2). In addition to being separated from other *S*. Montevideos associated with the spiced-meat contamination event by nine phylogenetically informative SNPs (Figure 4A), comparative analysis revealed the presence of a 100 kb insertion with substantial homology to Enterobacterial phage D6. Since phage D6 was incomplete in GenBank (No. AY753669), a MAUVE comparison to another homologous relative, phage P1 (No. NC_005856), was helpful in suggesting that this may represent a D6-like phage insertion into contig 104 in this particular *S*. Montevideo genome. Based on the known length of phage D6, this particular insertion in *S*. Montevideo strain 157 accounts for observed variation between this genome (~ 4.75 Mb) and the other spiced-meat *S*. Montevideo genomes reported here (~ 4.65 Mb). Moreover, this finding underscores the utility of whole-genome scanning technologies for placing the source of size polymorphisms between otherwise homogeneous strains of *Salmonella*.

### NGS reveals phylogenetic discordance of hyper-discriminatory PFGE enzymes in an *S*. Montevideo outbreak cluster

The extent of phylogenetically congruent clustering between NGS and other conventional subtyping technologies such as PFGE, MLST, or MLVA is largely unknown for most serovars of *S. enterica*. Congruence is important in accessing the ability of subtyping methods to accurately assign genetic relatedness among closely related strains, such as those implicated in foodborne outbreak events [31]. Previous studies from our laboratory and elsewhere have demonstrated enhanced discrimination and accuracy for PFGE in assigning genetic relatedness of some *Salmonella *and *E. coli *O157:H7 strains by concatenating up to six different restriction enzyme patterns into single cluster analyses [6,10,31,32].

The availability of whole-genome sequences of *Salmonella*, such as *S*. Montevideo, enables a comparison between the conclusions of an epidemiological investigation and the linked clusters obtained from comparative genomics of the suspect isolates. One can also examine the patterns of linkage based on other genetic tools to the epidemiological evidence such as the discriminatory power of several non-conventional PFGE enzymes in the highly homogeneous group of *S*. Montevideos described above. After generating PFGE patterns for the six enzymes reported previously as part of the published concatenated PFGE protocol for non-typhoidal *Salmonella *in a previous study of *S*. Enteritidis and *S*. Typhimurium [6], we overlaid individual enzyme patterns onto the *S*. Montevideo NGS tree presented in Figure 3 and assessed congruence (i.e. agreement) in cluster assignments between the two methods. Owing to the extreme genetic homogeneity among these strains, four of the six enzymes (*i.e., Xba*I, *Bln*I, *Spe*I, and *Sfi*I) revealed identical PFGE patterns for all 40 of the *S*. Montevideo isolates included in the whole-genome tree. Moreover, the predominant *pac*I pattern varied in only one isolate (*S*. Montevideo 211) from Chinese Perch. In contrast, however, *Not*I, an enzyme reported previously as having a high discriminatory index for *S*. Typhimurium and *S*. Enteritidis [6], yielded 18 distinct patterns among the 40 *S*. Montevideos comprising this outbreak cluster. Albeit, side-by-side comparison of *Not*I pattern variants with NGS subgroups delineated in the clone tree revealed evidence for homoplasy (*i.e.*, convergent pattern evolution) for this enzyme (Figure 3). That is, *NotI *patterns FDA.NotI.009, -.010. and -.011 were represented by *S*. Montevideo isolates from different subgroups in the tree suggesting that these patterns emerged independently in distinct places during the recent evolution of these isolates. As an example, pattern FDA.NotI.009 is represented twice in group A, once in group B and D, three times in group E, and four times in group F. Thus, while the concatenation of multiple PFGE enzyme data sets may permit a more accurate clustering of closely related *Salmonella*e, these data sound a cautionary note when attempting to cluster outbreak strains based on any single PFGE enzyme, including highly polymorphic ones such as *Not*I.

### Biological, laboratory, and technical replicates of *Salmonella *Montevideo support reproducibility of NGS applications

As the power of NGS is realized in public health settings, deployment of the technology is expected to become more commonplace. Thus, it is important to further evaluate the technology, addressing questions concerning expected variation between closely related strains, background variation, and SNP variation that may arise during sub-culturing possibly obscuring an accurate molecular epidemiological analysis of isolates associated with contamination or outbreak events. That is, does variation arise in subsequent passages of an isolate and, if so, can phylogenetic analysis overcome the potentially misleading background noise associated with this level of sensitivity? We investigated this issue by sequencing to ~15× coverage 11 *S*. Montevideo isolates derived from clinical-food matches associated with the 2009 spiced-Italian style meat contamination event, such that isolates were taken from the patient as well as the suspected corresponding food vehicle that sickened that particular patient. Specifically, we included seven matching *S*. Montevideo isolates from a single clinical/food source in Iowa and five isolates from a single clinical/food source in Connecticut. Additionally, we sequenced a single *S*. Montevideo food isolate (237_Lunch_Meat) for separate passages (4X) (*i.e.*, biological variation), separate colonies from the same passage (4X) (*i.e*, laboratory variation), and separate sequencing reactions from a single colony (4X) (*i.e*, Roche technical variation). Passages were conducted as follows: the initial sample was taken from frozen stock and plated on a TSA plate. Once plated it was incubated overnight at 37 degrees C. This was followed by Day 2 were sample was taken from the Day 1 overnight plate to inoculated the day 2 TSA plate. This day 2 plate was incubated overnight at 37 degrees C. Day 3 sample was taken from the Day 2 overnight plate and inoculated a day 3 TSA plate which was then incubated overnight at 37 degrees C. Day 4 sample was from the Day 3 overnight plate to inoculated the day 4 TSA plate and incubated overnight at 37 degrees C. After each plate was grown overnight, growth was taken from that plate and grown up a broth culture for DNA extraction of each of the genomic samples. Also, all samples were not single colony isolates for any of these plates. All passages and samples are representative cultures from the full plate and not just single colony.

Whole-genome sequencing yielded an alignment of approximately 4.5 mbps for downstream analysis. A total of 639 variable SNP sites were identified of which 23 were found to be parsimony informative among the validation isolates described above. However, once the data filter was applied to the remaining SNPs (*i.e.*, elimination of SNPs in homopolymeric tracts, adjacent to assembly breakpoints, and duplicated in other lineages), only a single informative SNP at position 3,823,524 was found remaining which was stable in the original *S*. Montevideo isolate (237_Lunch_Meat_IA_1) and all of its downstream genomes derived from subsequent passages, colonies, and DNA samples of this one strain (Table 4). We also searched for SNPs using the proprietary run Mapping software from Roche and found the SNP corresponding to position 3,823,524 in the WGS alignment (results not shown).

**Table 4:** Variable SNP calls discovered with resequencing and results after these were passed through our data filter.

Position	Description
204781	Missing after MUSCLE
255578	Homopolymer (8 T/A)
355131	Missing after MUSCLE
756435	Homopolymer (9 C/G)
1070504	SNP in Gap
1097814	Homopolymer (9 T/A)
1179704	Homopolymer (7 T/A)
1205130	Good (but ambiguous after looking at assembly)
1368882	Missing after MUSCLE
1642240	Missing after MUSCLE
1693620	Homopolymer (6 T/A)
1713322	Missing after MUSCLE
1806153	Homopolymer (6 T/A)
2087876	Missing after MUSCLE
2354057	Homopolymer (6 T/A)
2545225	Missing after MUSCLE
3193883	Homopolymer (7 T/A)
3823524	Good
4257557	Homopolymer (8 T/A)
4545198	SNP in Gap
4545878	Duplicated in other Salmonella (Elongation Factor Tu)
4546413	SNP in Gap
4548105	23S rRNA

Details of the variation arising from resequencing are listed including the variable site location, a description of the variant, the nucleotide change and corrections during our rapid data analysis pipeline.

**Figure 5:**
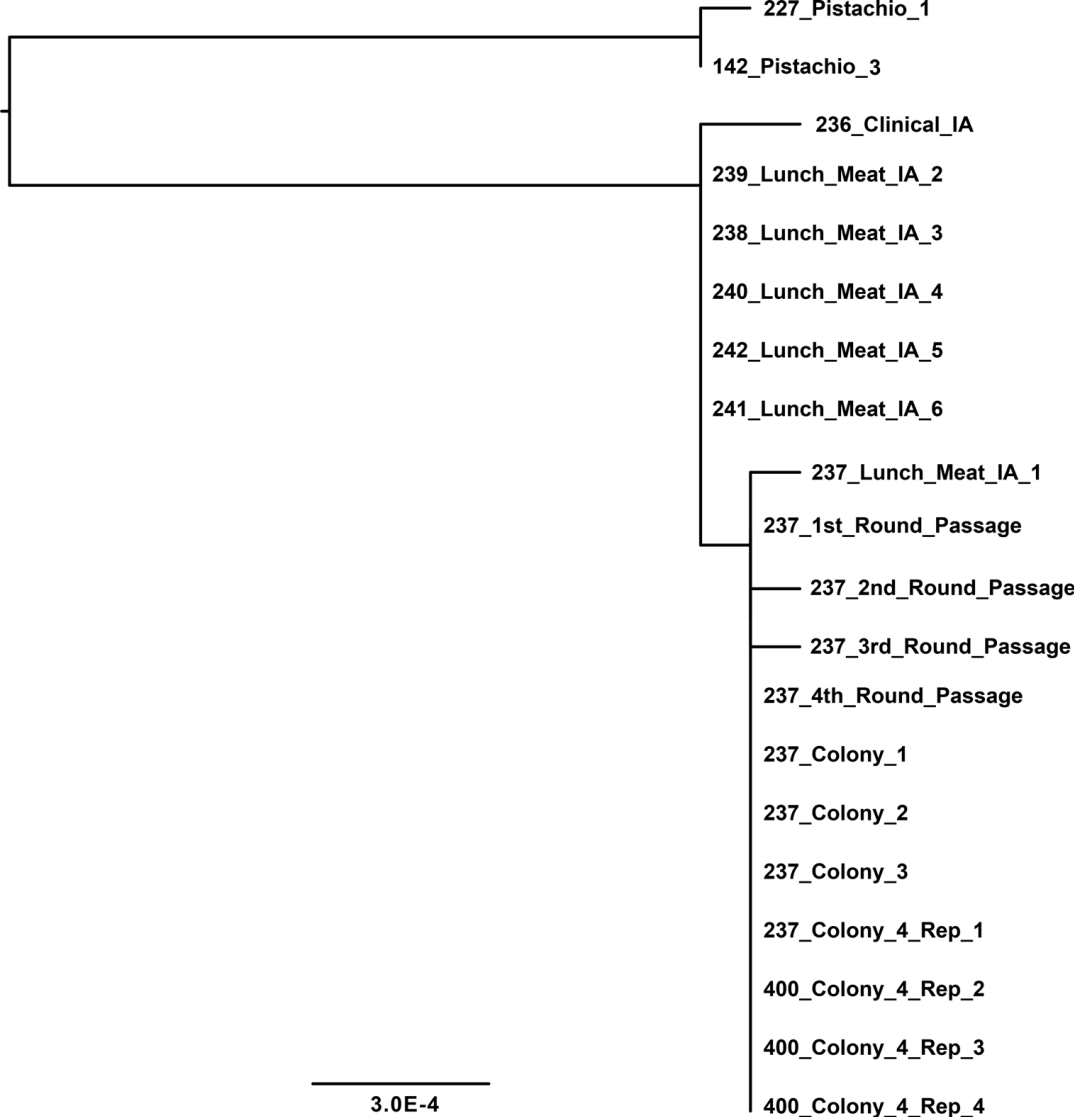
**Phylogenetic GARLI tree from resequencing of matching human-food isolate pairs, individual colonies, and sub-passages of a single strain of *S*. Montevideo**. Terminal names, scale bar, and branch lengths are as in Figure 1. The tree was rooted with two outgroup isolates, both of which were obtained from Pistachio. The laboratory-generated isolates were indistinguishable in a phylogenetic analysis with all replicates clustering together. Some of the biological, laboratory, or technical replicate genomes yielded nucleotide differences and these are seen as longer terminal branches for several isolates on the tree. These few changes did not alter relatedness or inclusivity/exclusivity among the matching food/human isolates.

As expected, these laboratory-generated isolates were indistinguishable in a phylogenetic analysis with the single parsimony informative SNP separating the 237_Lunch_Meat_IA_1 *S*. Montevideo isolate series from the other Iowa matching clinical-food isolates (Figure 5). Among the replicate genomes, only two sequences, genomes from *S*. Montevideo isolate 237 from the second and third round passages, retained actual SNP variation that emerged on the tree. That is, save for a single nucleotide difference present in the original 237 sample and two of the four downstream passages (*i.e.*, 237-second round and 237-third round), none of the additional biological, laboratory, or technical replicate genomes yielded nucleotide differences after alignment and quality filtration. It is important to note that these few changes did not alter relatedness or inclusivity/exclusivity among the matching food/human isolates. Rather, the only structural difference in the tree to arise from these three changes was in the form of branch length for the individual isolates affected. Additionally, it is noteworthy that, in the larger outbreak clone tree, the Connecticut and Iowa matching isolates were both phylogenetically inseparable from their sister isolates, and collectively, both strain sets sorted squarely among the spiced-meat food, environmental, and clinical isolates associated with the same contamination event (Figure 3). These findings indicate that when NGS data are quality filtered and inspected carefully using inclusivity/exclusivity criteria, the resultant stable and informative SNP data can be used effectively to phylogenetically partition closely related isolates of *S. enterica *(*i.e., S*. Montevideo). Consistent with this find, a bolus of successful applications is now accruing [17-20].

### NGS provides discovery for development of novel MLVA targets

Aside from its emerging and direct role in highly homogeneous *S*. enterica outbreaks, it is important to recall the important function of NGS for augmenting conventional detection, identification, and subtyping methods development. Currently, several rapidly evolving regions of the *Salmonella *chromosome are under investigation for their utility for enhanced subtyping of highly homogeneous *Salmonella *strains associated with foodborne outbreaks. Specifically, select VNTRs (variable number tandem repeats) in the genomes of *Salmonella *and *E. coli *have been targeted to develop markers and probes for MLVA (multi-locus VNTR analysis), a rapid and sensitive subtyping method that fingerprints the genomes of closely related strains based on size polymorphism of VNTR sequences [33,34].

Since MLVA protocols are developed at the serovar level for *Salmonella*, very few are available save for the most significant and widely studied *Salmonella*e (*i.e., S*. Typhimurium and *S*. Enteritidis). Here, using our NGS alignments only, we identified a polymorphic VNTR region within *S*. Montevideo that may serve in the development of a MLVA protocol for this important foodborne serovar as well (Table 5). The locus was identified within a cell division gene (*fts*N) and delineates the major *S*. Montevideo lineages represented in our NGS serovar tree in Figure 1. Moreover, this finding illustrates the importance of providing NGS data from multiple strains and multiple serovars in order to foster the identification of additional MLVA loci to support rapid subtyping protocols for *Salmonella *serovars of public health significance.

**Table 5:** Polymorphic VNTR discovery found within a cell division gene (*fts*N) in *S*. Montevideo using NGS applications.

160_Clinical_FL	TGCGTTTGAGCCCACTGCTGCTGCTGCTGCTGCTGCTGCTGCTGCTGCTGCTGCTGCGCCT
206_Clinical	TGCGTTTGAGCCCA-----------------------------CTGCTGCTGCTGCTGCTGCTGCTGCGCCT
161_Clinical_1993	TGCGTTTGAGCCCA-----------------------------CTGCTGCTGCTGCTGCTGCTGCTGCGCCT
207_Sunflower	TGCGTTTGAGCCCA-------------------------------------CTGCTGCTGCTGCTGCTGCGCCT
205_Soup	TGCGTTTGAGCCCA-----------------------------------------CTGCTGCTGCTGCTGCGCCT
162_Reference	TGCGTTTGAGCCCA-----------------------------------------CTGCTGCTGCTGCTGCGCCT
163_Clinical_GA	TGCGTTTGAGCCCA-----------------------------------------CTGCTGCTGCTGCTGCGCCT
221_Clinical_NC_2	TGCGTTTGAGCCCA---------------CTGCTGCTGCTGCTGCTGCTGCTGCTGCTGCTGCGCCT
223_Clinical_NC_1	TGCGTTTGAGCCCA---------------CTGCTGCTGCTGCTGCTGCTGCTGCTGCTGCTGCGCCT
222_Clinical_NC_5	TGCGTTTGAGCCCA---------------CTGCTGCTGCTGCTGCTGCTGCTGCTGCTGCTGCGCCT
220_Clinical_NC_3	TGCGTTTGAGCCCA---------------CTGCTGCTGCTGCTGCTGCTGCTGCTGCTGCTGCGCCT
217_Drain_Swab	TGCGTTTGAGCCCA---------------CTGCTGCTGCTGCTGCTGCTGCTGCTGCTGCTGCGCCT
155_Clinical_NC_4	TGCGTTTGAGCCCA---------------CTGCTGCTGCTGCTGCTGCTGCTGCTGCTGCTGCGCCT
227_Pistachio_1	TGCGTTTGAGCCCA---------------CTGCTGCTGCTGCTGCTGCTGCTGCTGCTGCTGCGCCT
212_King Fish	TGCGTTTGAGCCCA---------------CTGCTGCTGCTGCTGCTGCTGCTGCTGCTGCTGCGCCT
204_Chicken	TGCGTTTGAGCCCA---------------CTGCTGCTGCTGCTGCTGCTGCTGCTGCTGCTGCGCCT
147_Black_Pepper3	TGCGTTTGAGCCCA---------------CTGCTGCTGCTGCTGCTGCTGCTGCTGCTGCTGCGCCT
148_Black_Pepper4	TGCGTTTGAGCCCA---------------CTGCTGCTGCTGCTGCTGCTGCTGCTGCTGCTGCGCCT
142_Pistachio_2	TGCGTTTGAGCCCA---------------CTGCTGCTGCTGCTGCTGCTGCTGCTGCTGCTGCGCCT
224_Clinical_OH_2	TGCGTTTGAGCCCA---------------CTGCTGCTGCTGCTGCTGCTGCTGCTGCTGCTGCGCCT
216_Black_Pepper2	TGCGTTTGAGCCCA---------------CTGCTGCTGCTGCTGCTGCTGCTGCTGCTGCTGCGCCT
215_Red_Pepper_2	TGCGTTTGAGCCCA---------------CTGCTGCTGCTGCTGCTGCTGCTGCTGCTGCTGCGCCT
158_Clinical_MD	TGCGTTTGAGCCCA---------------CTGCTGCTGCTGCTGCTGCTGCTGCTGCTGCTGCGCCT
225_Clinical_OH_1	TGCGTTTGAGCCCA---------------CTGCTGCTGCTGCTGCTGCTGCTGCTGCTGCTGCGCCT
228_Clinical_CT	TGCGTTTGAGCCCA---------------CTGCTGCTGCTGCTGCTGCTGCTGCTGCTGCTGCGCCT
229_Salami_2_CT	TGCGTTTGAGCCCA---------------CTGCTGCTGCTGCTGCTGCTGCTGCTGCTGCTGCGCCT
230_Salami_1_CT	TGCGTTTGAGCCCA---------------CTGCTGCTGCTGCTGCTGCTGCTGCTGCTGCTGCGCCT
233_Salami_CT	TGCGTTTGAGCCCA---------------CTGCTGCTGCTGCTGCTGCTGCTGCTGCTGCTGCGCCT
235_Salami_CT	TGCGTTTGAGCCCA---------------CTGCTGCTGCTGCTGCTGCTGCTGCTGCTGCTGCGCCT
214_Black_Pepper1	TGCGTTTGAGCCCA---------------CTGCTGCTGCTGCTGCTGCTGCTGCTGCTGCTGCGCCT
213_Sea_Trout	TGCGTTTGAGCCCA---------------CTGCTGCTGCTGCTGCTGCTGCTGCTGCTGCTGCGCCT
219_Red_Pepper_1	TGCGTTTGAGCCCA---------------CTGCTGCTGCTGCTGCTGCTGCTGCTGCTGCTGCGCCT
156_Clinical_OH_3	TGCGTTTGAGCCCA---------------CTGCTGCTGCTGCTGCTGCTGCTGCTGCTGCTGCGCCT
209_Romaine	TGCGTTTGAGCCCA---------------CTGCTGCTGCTGCTGCTGCTGCTGCTGCTGCTGCGCCT
237_Meat_IA_1	TGCGTTTGAGCCCA---------------CTGCTGCTGCTGCTGCTGCTGCTGCTGCTGCTGCGCCT
238_Meat_IA_3	TGCGTTTGAGCCCA---------------CTGCTGCTGCTGCTGCTGCTGCTGCTGCTGCTGCGCCT
239_Meat_IA_2	TGCGTTTGAGCCCA---------------CTGCTGCTGCTGCTGCTGCTGCTGCTGCTGCTGCGCCT
240_Meat_IA_4	TGCGTTTGAGCCCA---------------CTGCTGCTGCTGCTGCTGCTGCTGCTGCTGCTGCGCCT
242_Meat_IA_5	TGCGTTTGAGCCCA---------------CTGCTGCTGCTGCTGCTGCTGCTGCTGCTGCTGCGCCT
211_Perch	TGCGTTTGAGCCCA---------------CTGCTGCTGCTGCTGCTGCTGCTGCTGCTGCTGCGCCT
210_Mozzarella	TGCGTTTGAGCCCA---------------CTGCTGCTGCTGCTGCTGCTGCTGCTGCTGCTGCGCCT
236_Clinical_IA	TGCGTTTGAGCCCA---------------CTGCTGCTGCTGCTGCTGCTGCTGCTGCTGCTGCGCCT
157_Clinical_CA	TGCGTTTGAGCCCA---------------CTGCTGCTGCTGCTGCTGCTGCTGCTGCTGCTGCGCCT
144_Black_Pepper6	TGCGTTTGAGCCCA---------------CTGCTGCTGCTGCTGCTGCTGCTGCTGCTGCTGCGCCT
145_Black_Pepper5	TGCGTTTGAGCCCA---------------CTGCTGCTGCTGCTGCTGCTGCTGCTGCTGCTGCGCCT
146_Black_Pepper7	TGCGTTTGAGCCCA---------------CTGCTGCTGCTGCTGCTGCTGCTGCTGCTGCTGCGCCT

Terminal names correspond to samples in Table 1.

## Discussion

Here, we reported the use of NGS technology for describing the phylogenetic diversity of *S*. Montevideo, a significant serovar of *S*. enterica involved in numerous outbreaks and product recalls http://www.cdc.gov/ncidod/dbmd/phlisdata/Salmonella.htm#2009. Moreover, we have applied informative substitutions from these genomes to further ascertain phylogenetic relatedness among a highly homogeneous *S*. Montevideo clone, of which some strains were associated with a recent spiced-meat outbreak event in the U.S last year. In this instance, the investigatory utility of NGS became apparent as the unusual genetic homogeneity among both outbreak associated and non-associated *S*. Montevideo strains could not be resolved unambiguously with more conventional genotyping approaches. Comparative genomic molecular epidemiology produced hundreds of SNP differences across distinct lineages of *S*. Montevideo and even provided broad size differences among the most distantly diverged strains of this serovar. Among the *S*. Montevideos populating clade IV in the serovar tree, nearly all shared common pulsotypes for *Xba*I and *Bln*I as well as for several additional enzymes including *Spe*I, *Sfi*I, and *Pac*I. NGS combined with phylogenetic analysis, however, was able to delineate the scope of contamination by differentiating those strains associated with the spiced-meat outbreak from strains epidemiologically unrelated to this event despite the remarkable genetic identity linking these two strain sets. Given the extraordinary resolution that NGS provides--resolution best described as "nanotyping", it is not surprising that, when *Salmonella *isolates with divergent PFGE patterns are sequenced using NGS technology, the resultant alignments typically yield thousands of SNP differences.

For *S*. Montevideo, four disparate lineages of strains were observed (*i.e.*, clades I-IV, Figure 1). One lineage, in particular (*i.e.*, clade I in Figure 1), was characterized by a single isolate from sunflower, and it remained unclear as to whether the long branch distinguishing this isolate was due to changes that accumulated more recently from laboratory passages or whether observed variation in this strain accrued in a natural setting. Surprisingly, this sunflower isolate clustered with several *S*. Montevideos recently isolated from pet treats and a pet treat-manufacturing environment underscoring the potential risk associated with this and the other discrete lineages of this serovar http://www.fda.gov/NewsEvents/Newsroom/PressAnnouncements/ucm197700.htm. That is, it appears that foodborne contamination events can emerge from any of these diverged *S*. Montevideo lineages which are able to survive in foods and cause illness in humans. Clinical isolates were found in each of the major and separate lineages of *S*. Montevideo tested (Figure 1 Clades I, II, III, and IV). Moreover, such observations enforce the notion that in addition to these attributes, the risk to public health also stems from a particular *Salmonella *lineage simply gaining the opportunity to contaminate the human or animal food supply, rather than any one *S*. Montevideo lineage being more fit to persist in foods over any other.

Separation, based on SNP distances, among the four phyletic lineages of *S*. Montevideo reported here rivaled distances observed between *S*. Montevideo and other distinct *Salmonella *subspecies I serovars including *S*. Pomona, *S*. Javiana, and *S*. Schwarzengrund. Such remarkable interclade divergences suggests that the four major lineages of *S*. Montevideo diverged early in the evolution of this serovar, and each appears to have evolved largely independent of the others, an evolutionary pattern consistent with a hypothesis of unique host/niche adaptation for the separate lineages and sublineages that compose this serovar. This thesis is further supported by an examination of variable genes and SNPs that define various lineages of isolates within *S*. Montevideo clade IV. That is, 25 of 43 select informative SNPs (Table 3) defining subgroups within this clade were non-synonymous. Additionally, 78% of the representative informative SNPs clustering together two or more *S*. Montevideo subgroups were also found to be polymorphic. These data are reminiscent of a previous report by Soyer et al., [30] which noted a potentially significant role for positive selection based on an unusual proportion of non-synonymous substitutions across the genomes of several host-adapted serovars including *S*. Cholerasuis, *S*. Typhimurium, and the agent of Typhus, serovar *S*. Typhi (28). Taken together, these data signal *S*. Montevideo as a potentially niche-adapted and evolutionarily diverse serovar among the subspecies I *Salmonella*e, a conclusion additionally supported by an extraordinary ecological range and natural persistence in diverse environments (*e.g., S*. Montevideo has been found associated with spices, produce, poultry, beef and porcine commodities to name but a few).

The results from the genome validation study reported here also merit discussion. After collecting over 50 mbp of finished bacterial sequence for multiple downstream passages, colonies, and DNA preparations of a single *S*. Montevideo isolate, it was clear that NGS had provided sufficiently stable data to conclude that no single potential source of variability tested (*i.e.*, biological, laboratory or technical) was capable of altering phylogenetic conclusions uncovered during the comparative genomic investigation. That is, despite the detection of three substitutions among serially passaged genomes from a single *S*. Montevideo source, no re-sequenced replicate conflicted with our phylogenetic conclusions here or for strains included in a previously published letter defining an *S*. Montevideo spiced-meat outbreak cluster [5]. Rather, it is clear that phylogenetic approaches are providing rational and highly reproducible analytical outcomes for high-resolution NGS data pipelines and appear to be sufficiently robust for reconstructing strain relatedness based on the hundreds and sometimes thousands of informative changes that amass from a single NGS experiment.

Global deployment of NGS technology as a direct investigatory tool has already proven to be highly successful to the public health community. In addition to the NGS application described here for one non-typhoidal *Salmonella *serovar, NGS has provided extraordinary insight into case studies involving: (i) traceback of tuberculosis infections in Canada [20]; (ii) high-resolution evolutionary linkage of global clones of *Salmonella *Typhi [19]; and (iii) identification of the origins of the Haitian Cholera outbreak [17] as a few examples. It is important to note, however, that NGS data can provide additional utility for development of other subtyping methods. The MLVA locus presented here is one example of how NGS can serve as a genomic "compass" in seeking out VNTR regions with sufficient rates of change to develop custom MLVA assays for other important *Salmonella *serovars beyond *S*. Typhimurium and *S*. Enteritidis. Additionally, as shared NGS public health databases expand, many outbreak swarms will be defined by even more rapid and efficient re-sequencing protocols that target a subset of informative SNPs relative to the differentiation of a specific outbreak clone of pathogenic bacteria.

We would like to caution that the results reported here, while extremely encouraging, do not supplant the need for independent laboratory validations to establish SOPs for their particular platforms and chemistry and kits. Such validations may include the adoption of standard practices that have worked so well with past genetic testing, including CE methods that can more easily target and validate variable sites identified by whole genome sequencing and downstream phylogenetic analyses. Clearly, given the evolutionary rates governing nucleotide change among enteric bacteria combined with the risk of intrinsic polymerase error in the sequencing process itself, each step of the pathway, from isolate collection and template preparation to the sequencing reactions, could potentially spawn artifactual variability. A careful assessment of all of these sources of variation should provide more confidence for molecular epidemiological applications including the detection and scope of disease outbreak clusters.

## Conclusions

These results underscore the power of NGS, when coupled with phylogenetic analysis, to illuminate the genetic and evolutionary diversity of important serovars of *Salmonella enterica *along with the associated epidemiological pathways surrounding specific outbreak strains [17-20]. It appears that, at least in the case of *Salmonella*, the natural variation observed between strains is both stable and sufficient to allow for high resolution traceback of food and clinical isolates. It will be interesting to see whether ample genomic diversity can drive similar outcomes in other problematic taxa and highly clonal *Salmonella *serotypes. Moreover, NGS will provide the phylogenetic context on which to interpret other facile subtyping approaches that focus on more rapidly evolving genetic markers such as MLVA, rep-PCR, and CRISPRs [6-11,35] and will provide a novel suite of SNPs that will be critical to partitioning common *Salmonella *outbreak strains. In public health arenas, NGS strain "nanotyping" holds the potential to revolutionize the manner in which responses to outbreaks are managed. At a minimum, we see a future where NGS methods are brought to bare on the most difficult questions involving this enteric pathogen including direct application in foodborne outbreak cases in combination with other time-tested methods of epidemiologic investigation.

## Methods

### Data collection and analysis pipeline methods

Roche 454 GS Titanium NGS technology was employed in this study. This platform provided longer read lengths relative to other sequencers and a relatively shorter time to raw sequence [23]. Longer read lengths resulted in fewer contigs for draft assembly and aided in a more accurate placement of phage and plasmid sequences, both of which are commonplace among the group I salmonellae. All *S*. Montevideo isolates were draft shotgun sequenced using this platform and included 47 total isolates of *S*. Montevideo including 40 with PFGE patterns matching the spiced-meat outbreak (Figure 1, lineage IV) and 7 with unrelated PFGE patterns (Figure 1, lineages I-III). Additionally, 11 genomic replicates were sequenced for the validation experiment, including multiple colonies from the same plate (n = 4), multiple passages of an isolate (n = 4), and independent sequencing experiments (n = 4) from the same DNA source (Figure 5). Each isolate was run on a quarter of a titanium plate that produced roughly 250,000 reads per draft genome and coverage from 13× to 18×. Draft sequences for 34 of the 47 isolates were previously released as part of an outbreak case study [5] to test earlier hypotheses regarding the delimiting of foodborne contamination events.

De novo assemblies were created using the Roche Newbler (v 2.3) software package and the resulting contigs were annotated using NCBI's Prokaryotic Genomes Automatic Annotation Pipeline [PGAAP, [36]]. Phylogenetically informative SNP sites were identified using two independent alignment methods: 1) clustering of annotated open reading frames (ORFs) using reciprocal best Basic Local Alignment Search Tool [BLAST, http://blast.ncbi.nlm.nih.gov/Blast.cgi] hits with a 70% sequence identity setting followed by alignment with Multiple Sequence Comparison by Log-Expectation [MUSCLE, [37]], and 2) multiple genome alignment of WGS contigs using Mauve [38]. Duplicated genes were eliminated from all ORF clusters. The Mauve and ORF cluster alignments were then screened to find non-gap phylogenetically informative nucleotide positions (*i.e. *minor allele count ≥ 2). Informative positions from all ORF clusters and Mauve outputs were identical in the annotated protein coding regions. Informative positions for isolates in the outbreak cluster were manually checked to eliminate SNPs in homopolymers and repetitive elements. In this way, roughly 10-15 percent of the draft genome is filtered out, but the remaining SNPs are highly reproducible, providing sufficient variation for an informed molecular epidemiology interpretation [23].

Phylogenetic analysis of the clonal *S*. Montevideo data set including multiple serovars was performed on a set of 55,032 concatenated informative SNPs which encompasses the diversity within *S*. Montevideo. Approximately 99% of the sites in the 5 MB *Salmonella *genomes are phylogenetically uninformative and eliminating them dramatically reduces computation time and memory requirements. Phylogenetic analysis of the outbreak isolates was performed on the set of 43 concatenated ORFs containing informative SNPs. In all cases, phylogenetic trees were constructed using GARLI [39] under the maximum likelihood criterion. The phylogenetic tree in Figure 1 was constructed using GARLI under the GTR + gamma model of nucleotide evolution. The phylogenetic trees in Figure 3 and 5 were constructed using GARLI under the HKY + gamma model of nucleotide evolution.

The other related *Salmonella *including *S*. Schwarzendgrund and *S*. Javiana were taken from genbank (Table 1). *S*. Pomona was sequenced like the *S*. Montevideo isolates with an FDA ID number. One comparative genomics analysis suggested that *S*. Schwarzendgrund and *S*. Javiana are closely related [27] and our independent analyses, not shown, also would include *S*. Montevideo and *S*. Pomona in this cluster so we include all of these as outgroups.

We use the resultant phylogenetic trees to make hypotheses about the evolution of the *S*. Montevideo subtypes and the outbreak strains and to aid in investigation source tracking. We use these evolutionary hypotheses to identify reliable diagnostic nucleotide motifs (SNPs, rearrangements, and gene presences) for the identification of outbreak strains and for understanding the mechanisms that drive the outbreak occurrences. These methods allow both the rapid characterization of the genomes of foodborne pathogenic bacteria and can help identify the source of contamination of the food supply.

### Availability of data and cultures

All NCBI *S*. Montevideo genomes are linked to Bioproject 61937 which lists the new accession numbers AESR00000000-AESY00000000, AHIA00000000 and AHHT00000000 - AHHW00000000. Cultures included in this study are also available upon request to anyone with valid paper work and clearances. Please direct any queries to our strain curator Dwayne Roberson, at Dwayne.Roberson@fda.hhs.gov.

## Authors' contributions

ES and YL performed the bioinformatics analysis and analyzed the data; RS provided critical programming and bioinformatic tools; MWA, SMM, and EWB planned and conceived the experiments and co-wrote the manuscript; CL generated the sequencing libraries; and CEK and IS generated and analyzed the PFGE data. All authors read and approved the final manuscript.
